# Supplementary figures and images for: Archaeological expansions in tropical South America during the late Holocene: Assessing the role of demic diffusion
Source: PLoS One. 2020 Apr 27;15(4):e0232367. doi: 10.1371/journal.pone.0232367 (PMC7185720; doi:10.1371/journal.pone.0232367)

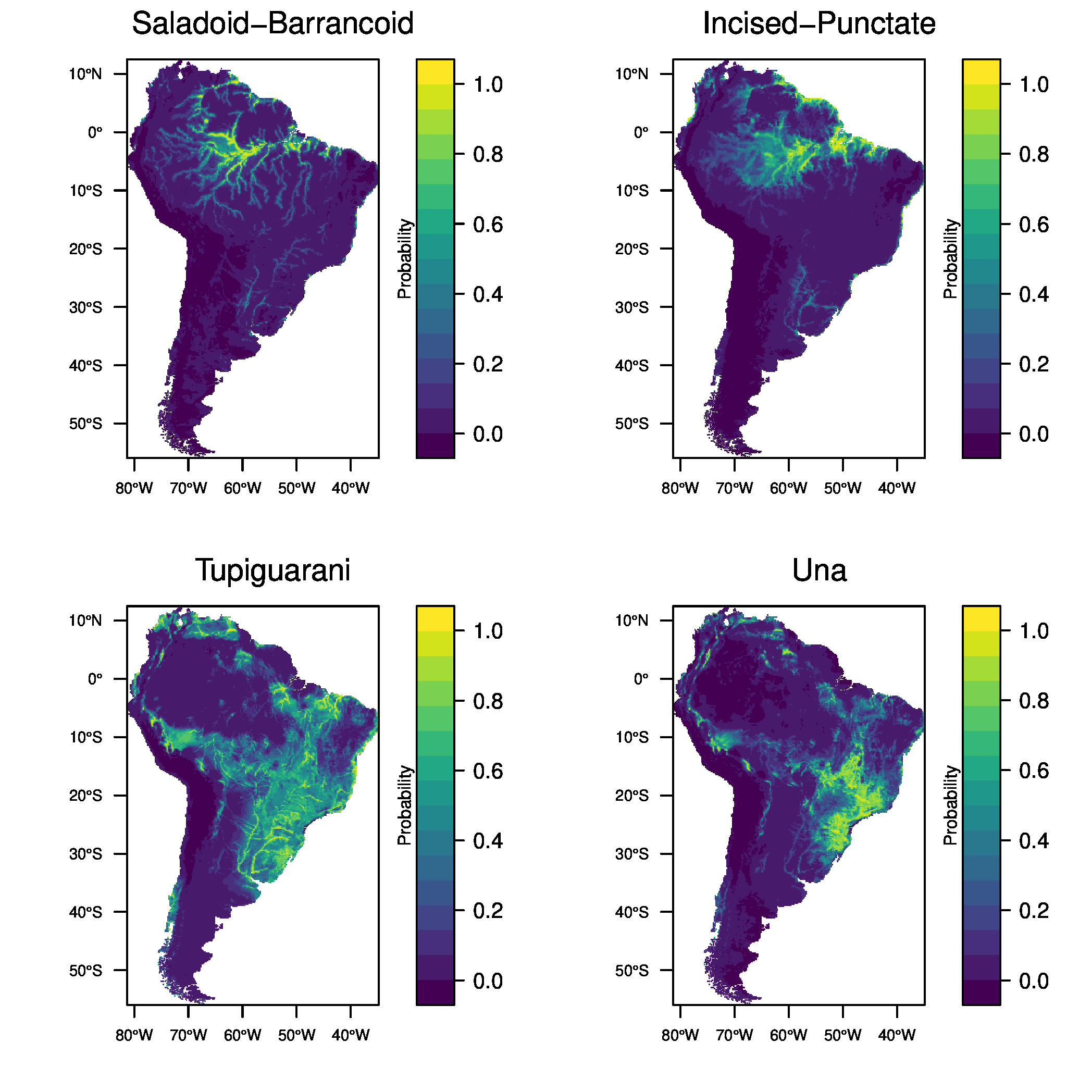

Supplement: S1 Fig — The scale of probability is given in the standard cloglog output of MaxEnt [119]. (TIFF) [file pone.0232367.s004.tiff]

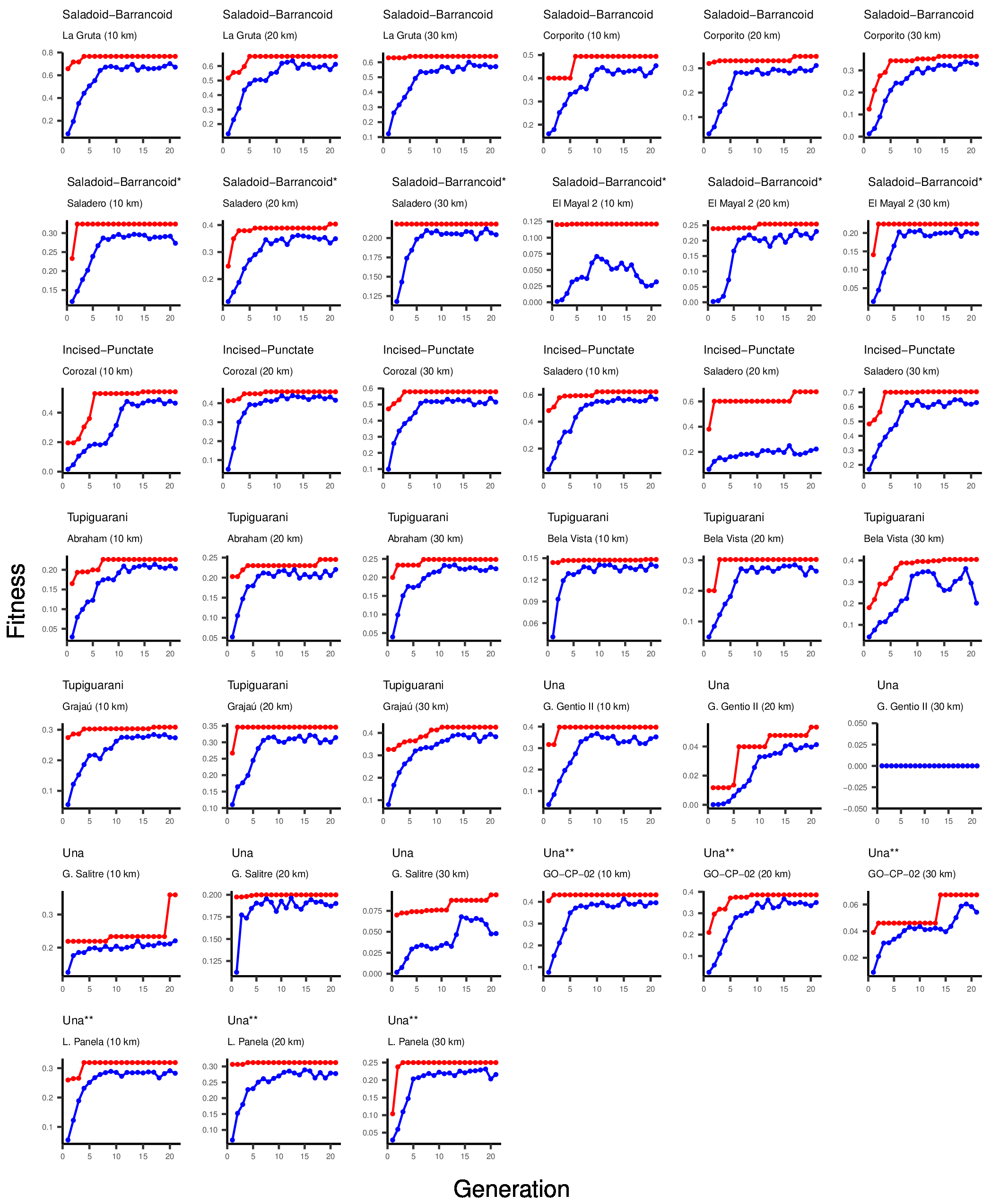

Supplement: S2 Fig — Blue and red lines are, respectively, the average and the maximum fitness of the population of models at each generation. *Models executed using the short chronology for the Orinoco. **Models executed without considering the earliest date for Gruta do Gentio II. (TIFF) [file pone.0232367.s005.tiff]

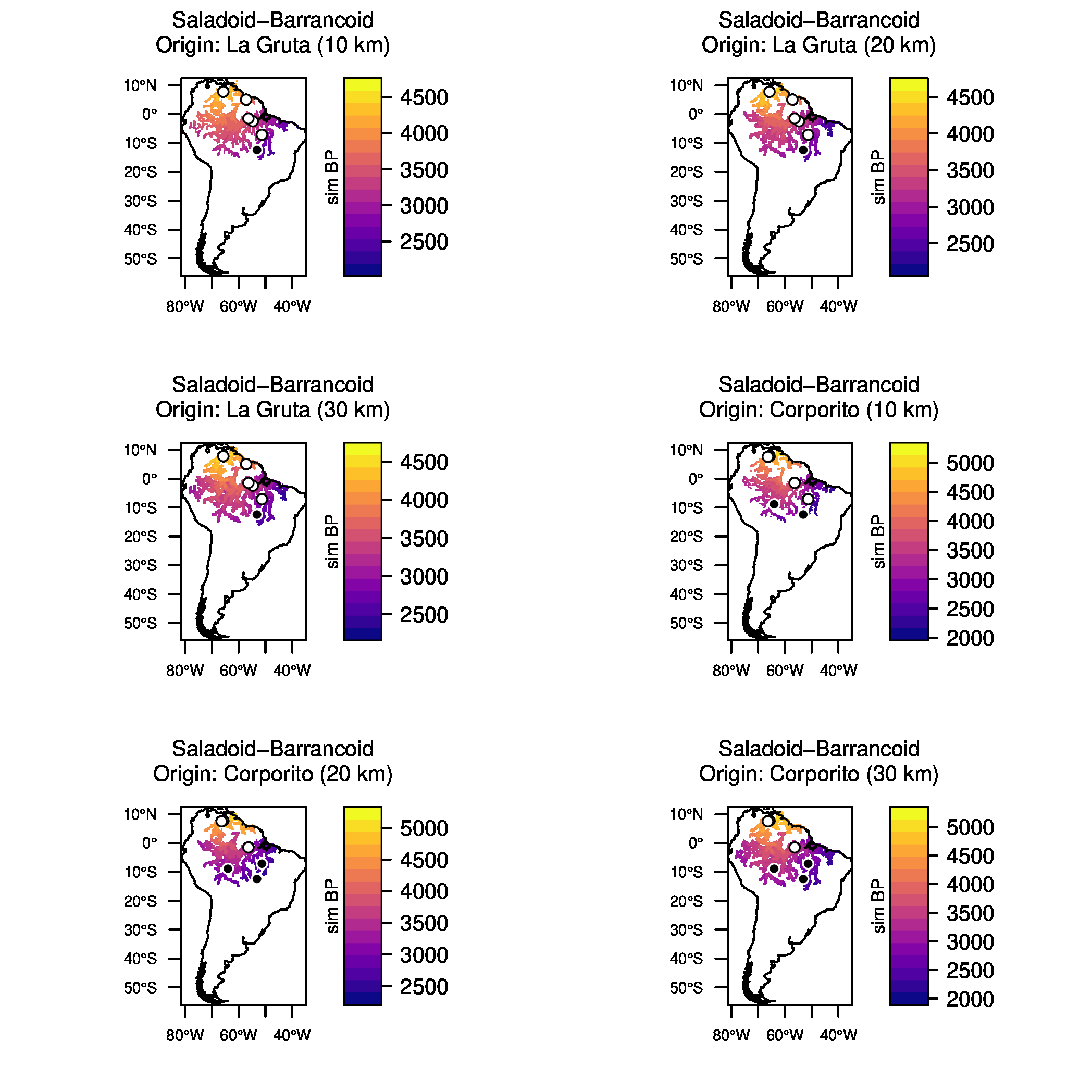

Supplement: S3 Fig — White circles are points where the simulated arrival date (sim BP) is within the 2σ interval of the respective calibrated 14C age, whereas black circles are points where the simulated arrival date is outside that range. (TIFF) [file pone.0232367.s006.tiff]

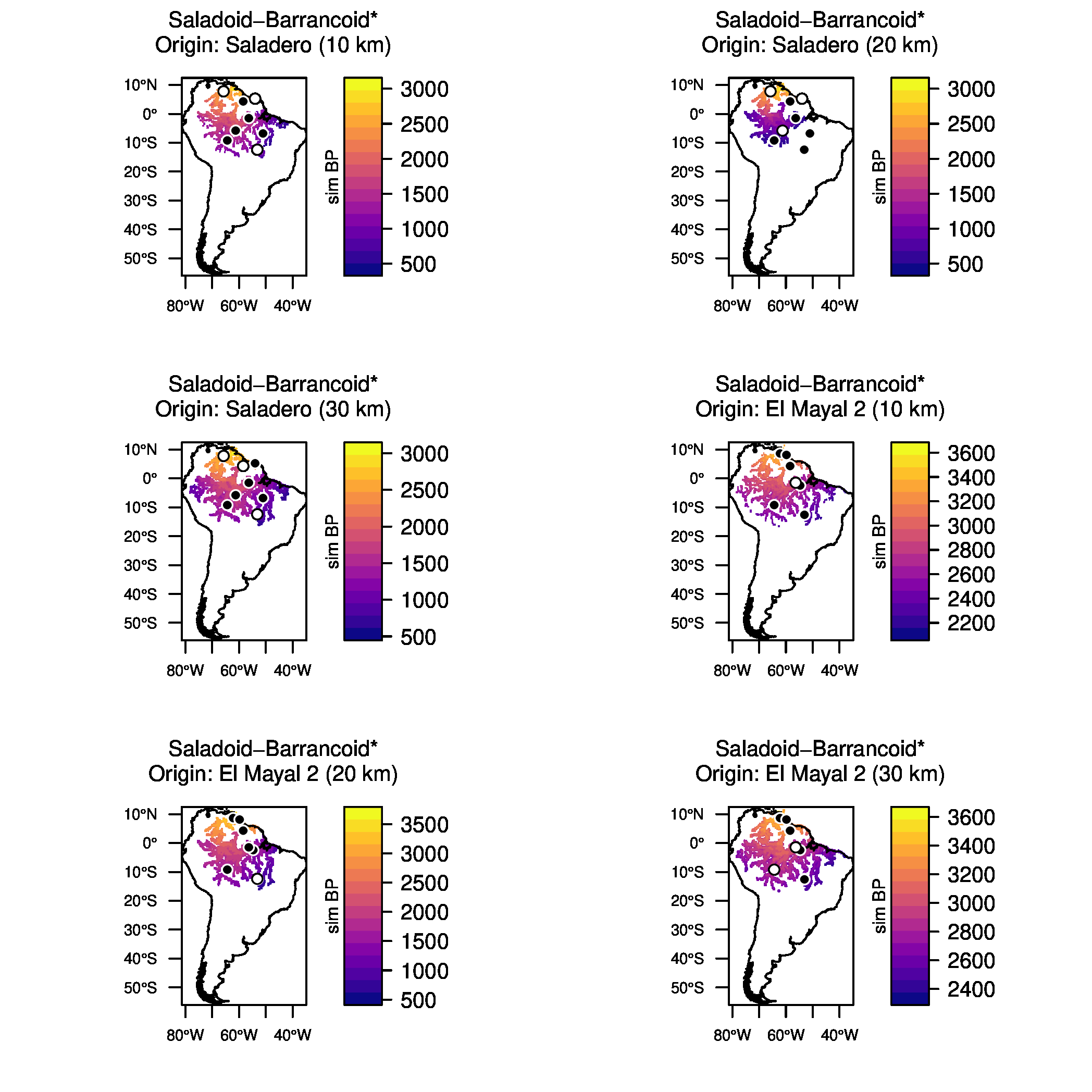

Supplement: S4 Fig — White circles are points where the simulated arrival date (sim BP) is within the 2σ interval of the respective calibrated 14C age, whereas black circles are points where the simulated arrival date is outside that range. *Models executed using the short chronology for the Orinoco. (TIFF) [file pone.0232367.s007.tiff]

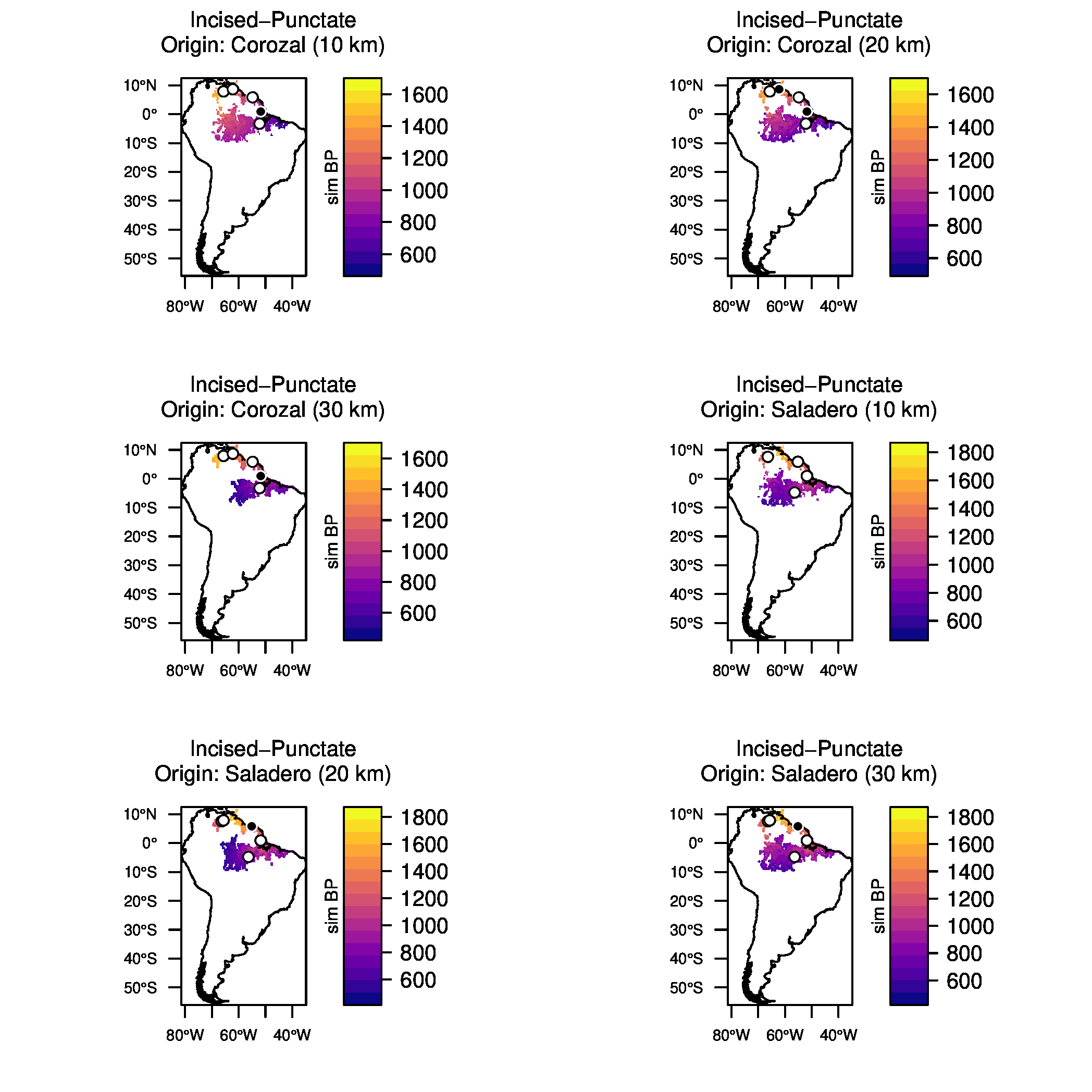

Supplement: S5 Fig — White circles are points where the simulated arrival date (sim BP) is within the 2σ interval of the respective calibrated 14C age, whereas black circles are points where the simulated arrival date is outside that range. (TIFF) [file pone.0232367.s008.tiff]

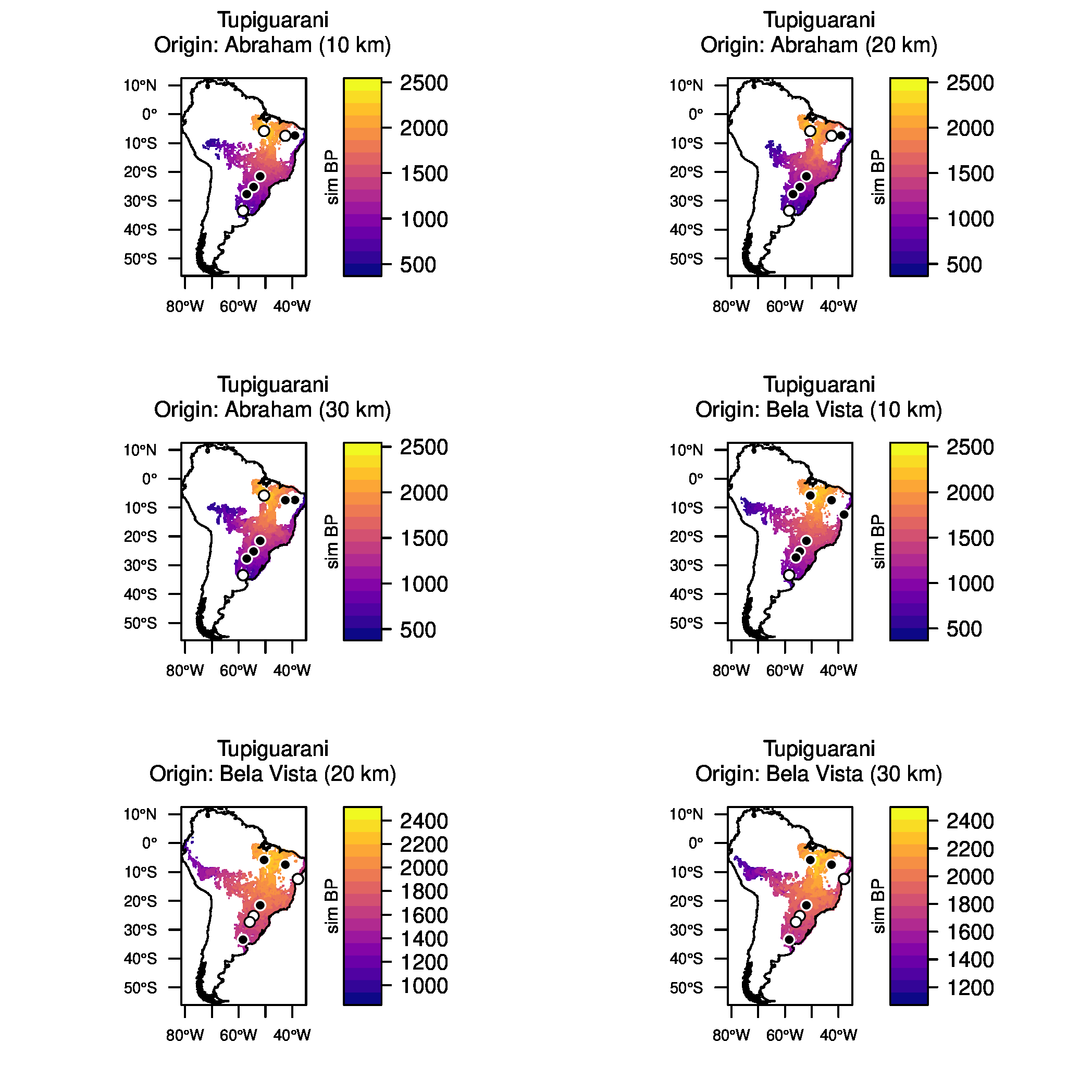

Supplement: S6 Fig — White circles are points where the simulated arrival date (sim BP) is within the 2σ interval of the respective calibrated 14C age, whereas black circles are points where the simulated arrival date is outside that range. (TIFF) [file pone.0232367.s009.tiff]

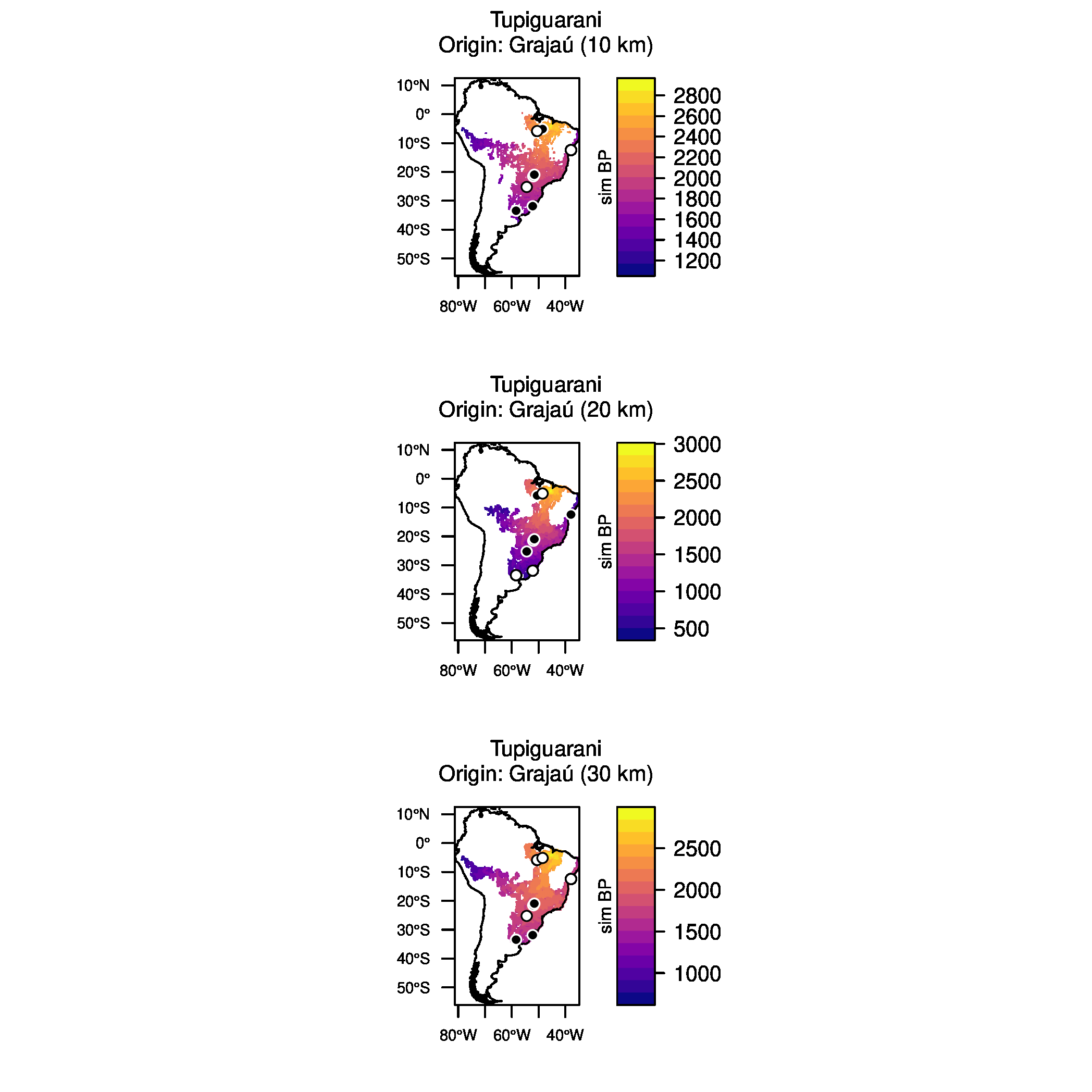

Supplement: S7 Fig — White circles are points where the simulated arrival date (sim BP) is within the 2σ interval of the respective calibrated 14C age, whereas black circles are points where the simulated arrival date is outside that range. (TIFF) [file pone.0232367.s010.tiff]

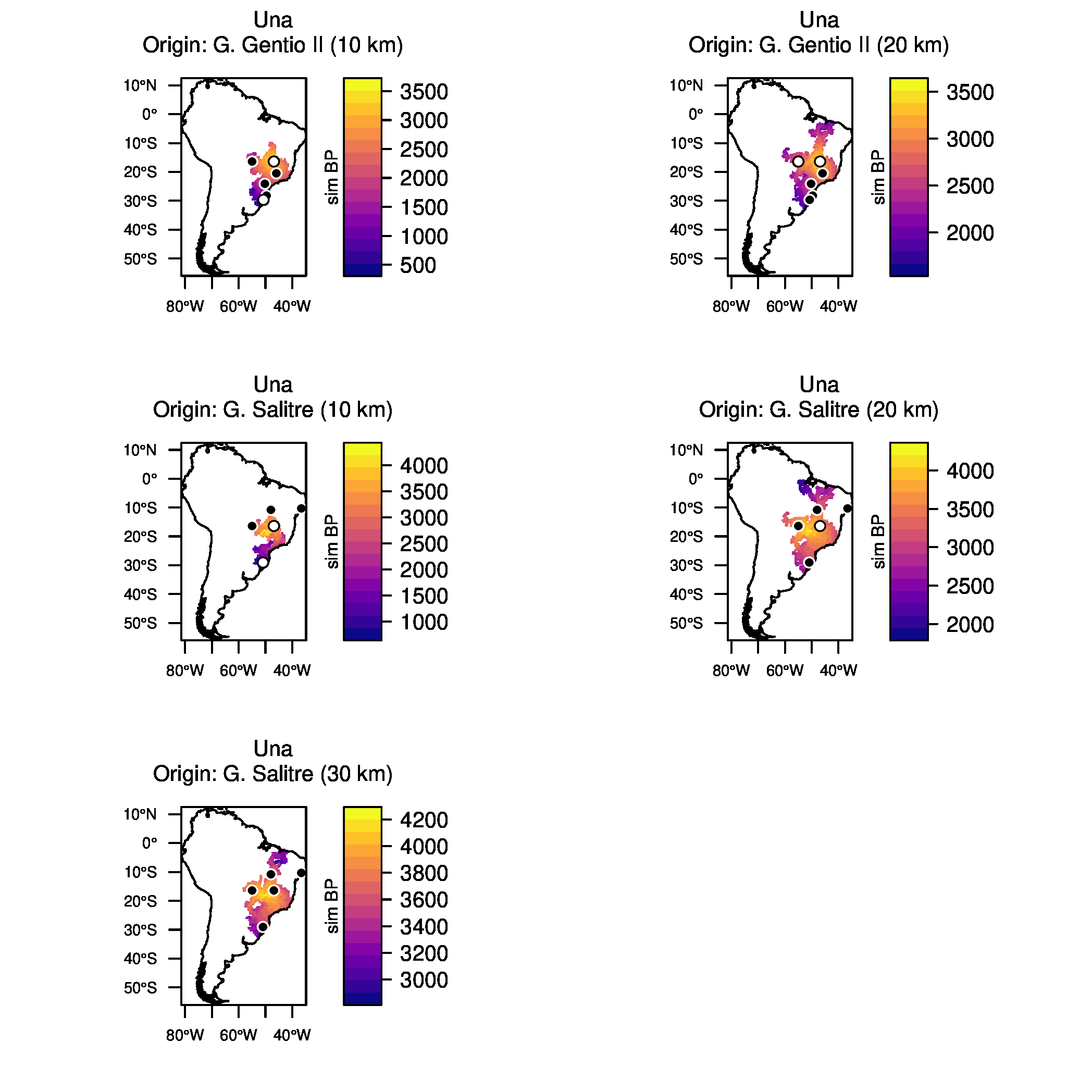

Supplement: S8 Fig — White circles are points where the simulated arrival date (sim BP) is within the 2σ interval of the respective calibrated 14C age, whereas black circles are points where the simulated arrival date is outside that range. (TIFF) [file pone.0232367.s011.tiff]

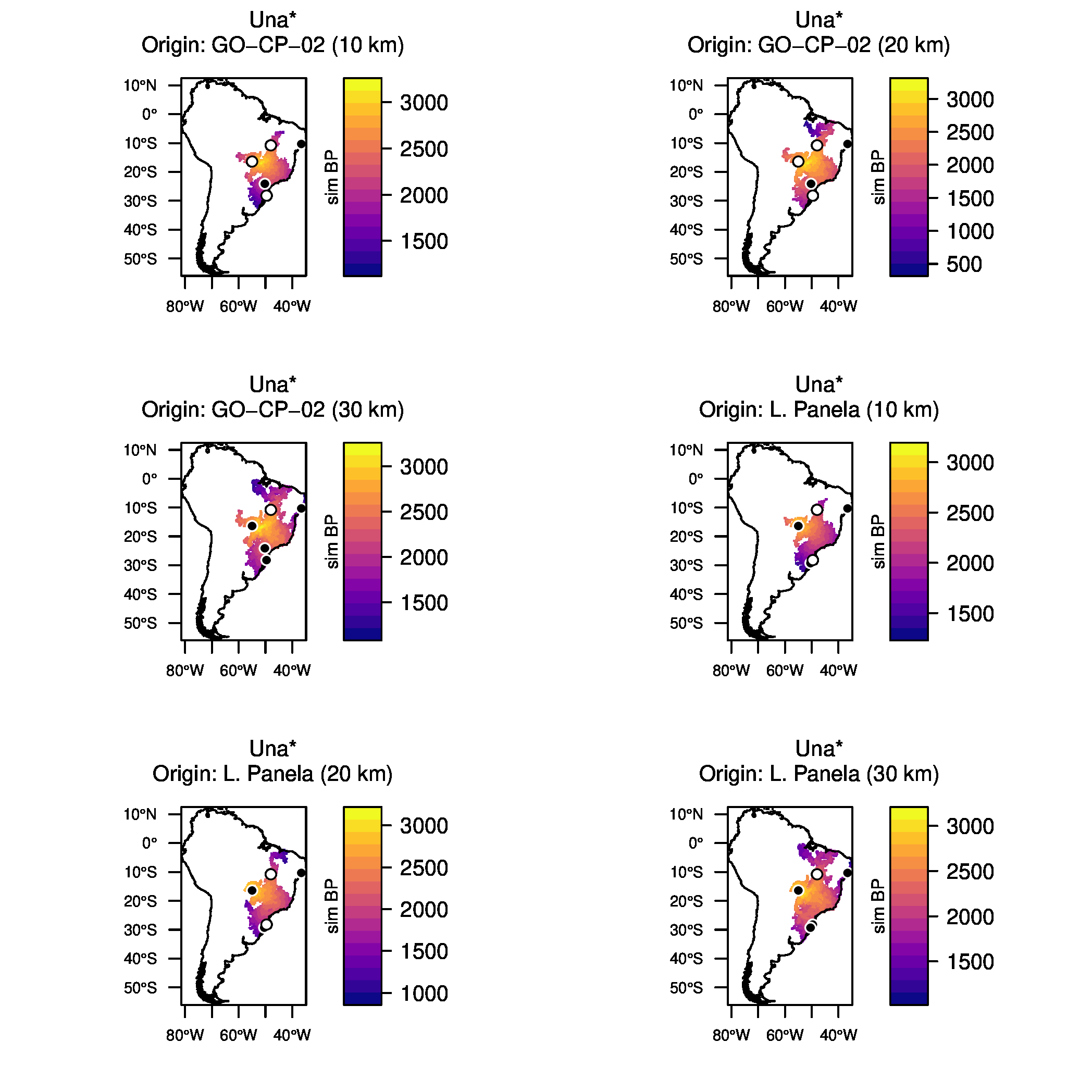

Supplement: S9 Fig — White circles are points where the simulated arrival date (sim BP) is within the 2σ interval of the respective calibrated 14C age, whereas black circles are points where the simulated arrival date is outside that range. *Models executed without considering the earliest date for Gruta do Gentio II. (TIFF) [file pone.0232367.s012.tiff]

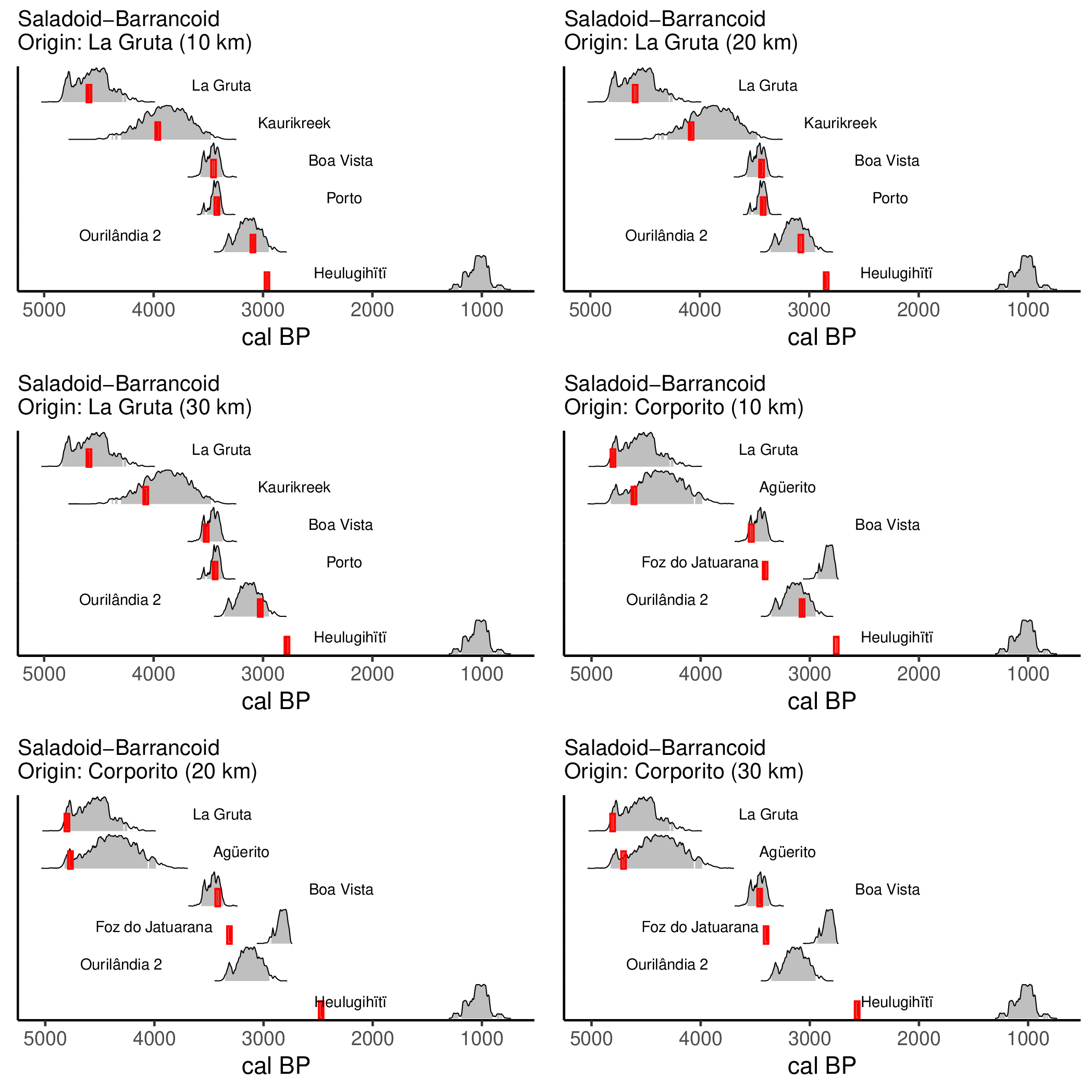

Supplement: S10 Fig — Shaded areas are the 2σ ranges of the calibrated probability densities of 14C archaeological dates, accompanied by the respective site name. Red bars represent the simulated arrival time. (TIFF) [file pone.0232367.s013.tiff]

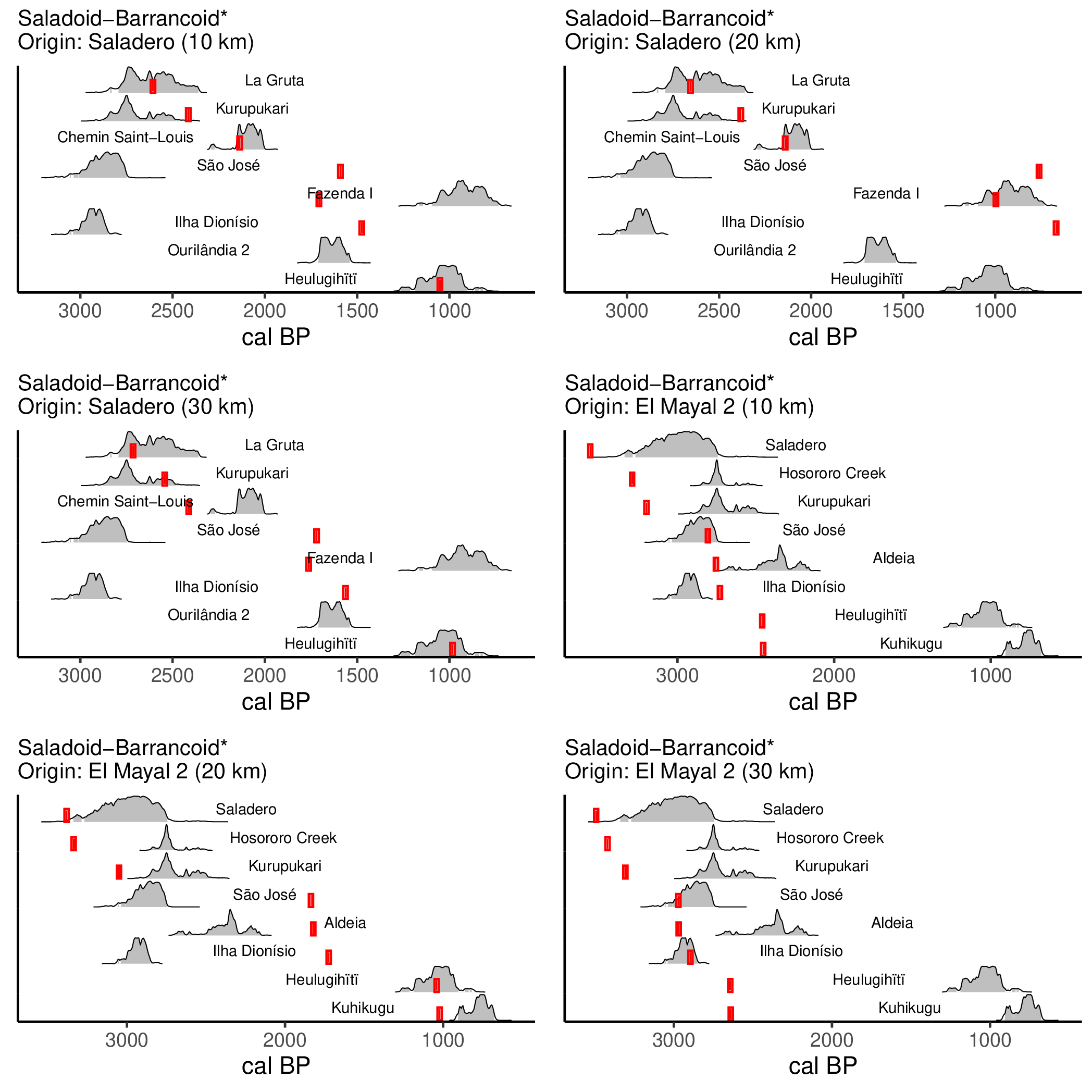

Supplement: S11 Fig — Shaded areas are the 2σ ranges of the calibrated probability densities of 14C archaeological dates, accompanied by the respective site name. Red bars represent the simulated arrival time. *Models executed using the short chronology for the Orinoco. (TIFF) [file pone.0232367.s014.tiff]

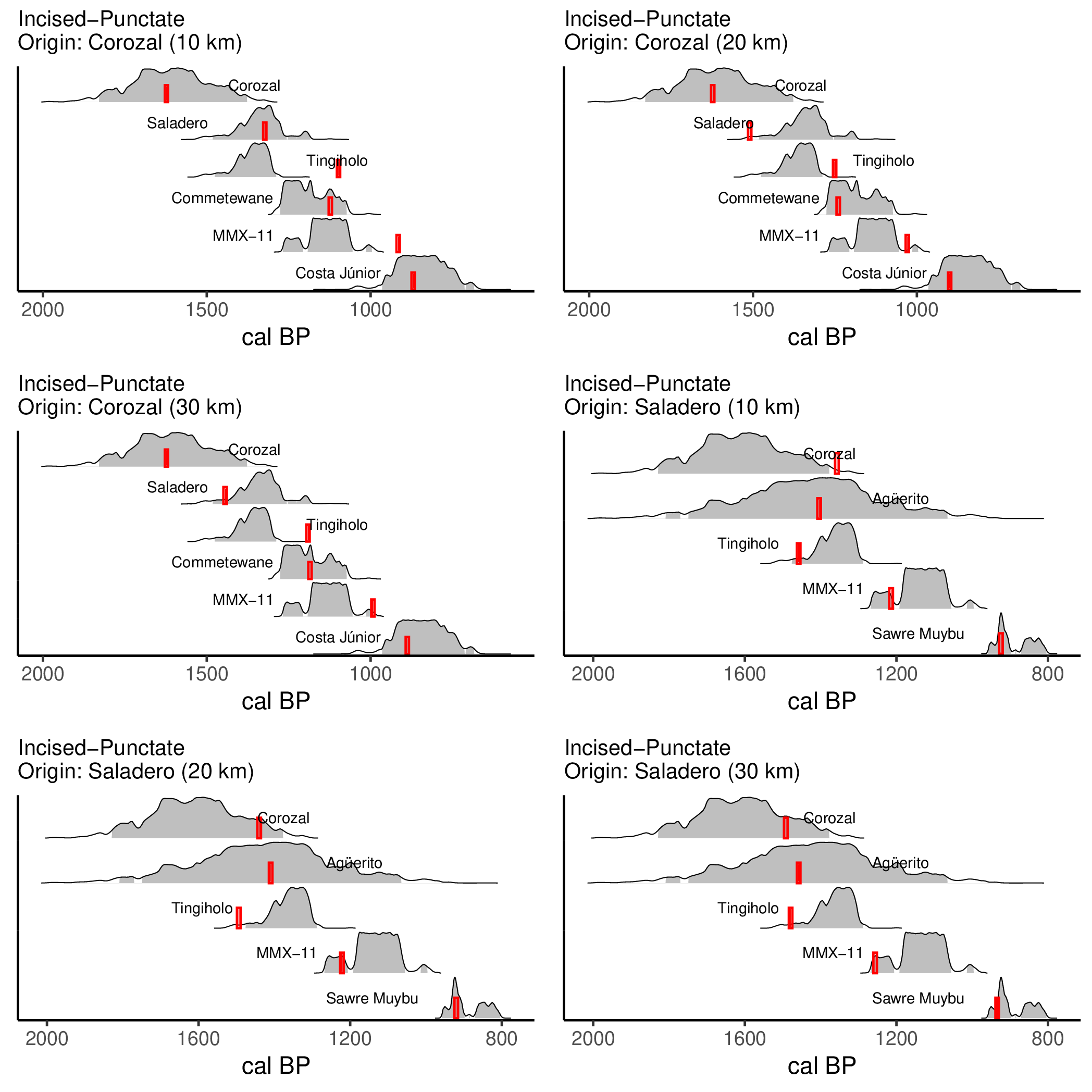

Supplement: S12 Fig — Shaded areas are the 2σ ranges of the calibrated probability densities of 14C archaeological dates, accompanied by the respective site name. Red bars represent the simulated arrival time. (TIFF) [file pone.0232367.s015.tiff]

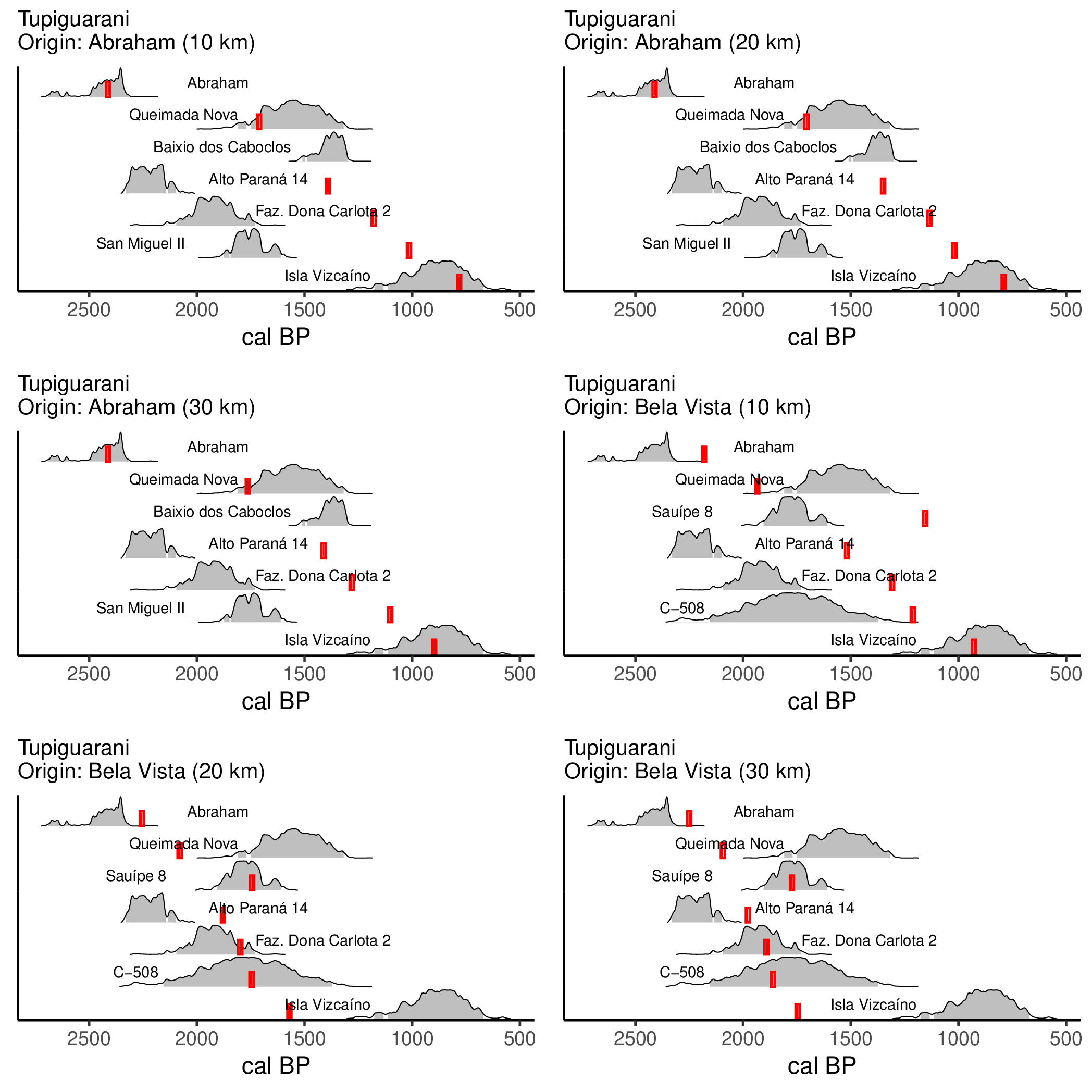

Supplement: S13 Fig — Shaded areas are the 2σ ranges of the calibrated probability densities of 14C archaeological dates, accompanied by the respective site name. Red bars represent the simulated arrival time. (TIFF) [file pone.0232367.s016.tiff]

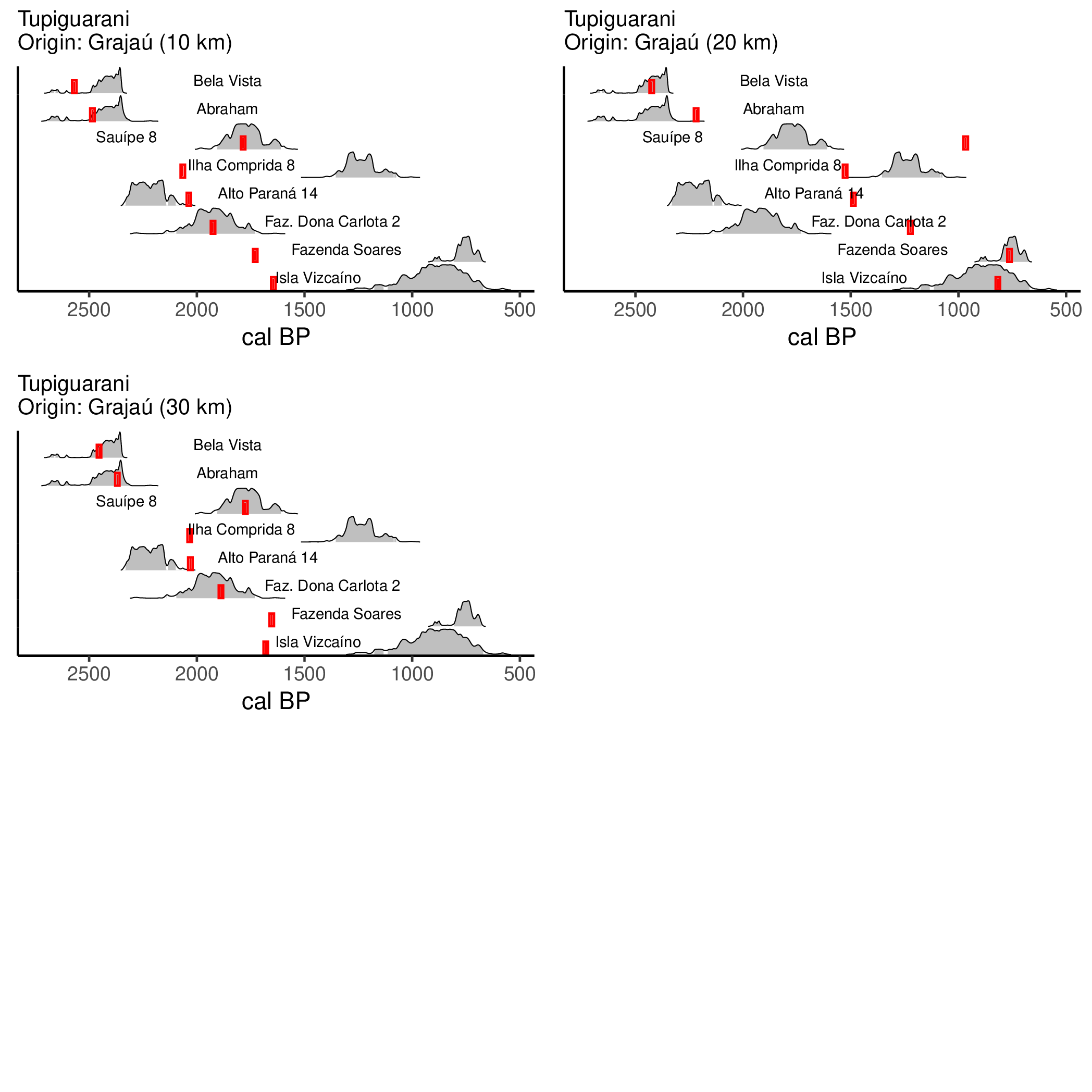

Supplement: S14 Fig — Shaded areas are the 2σ ranges of the calibrated probability densities of 14C archaeological dates, accompanied by the respective site name. Red bars represent the simulated arrival time. (TIFF) [file pone.0232367.s017.tiff]

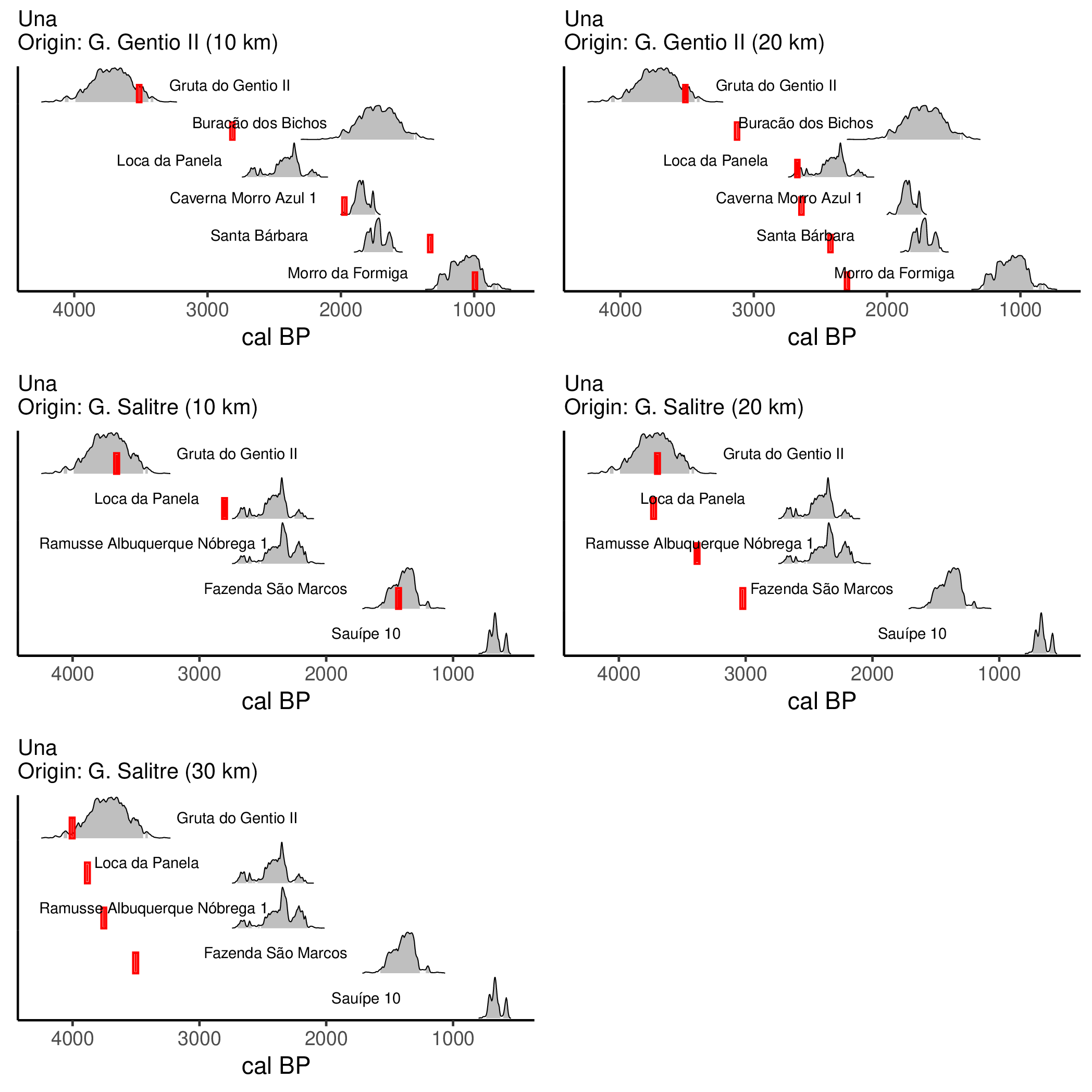

Supplement: S15 Fig — Shaded areas are the 2σ ranges of the calibrated probability densities of 14C archaeological dates, accompanied by the respective site name. Red bars represent the simulated arrival time. (TIFF) [file pone.0232367.s018.tiff]

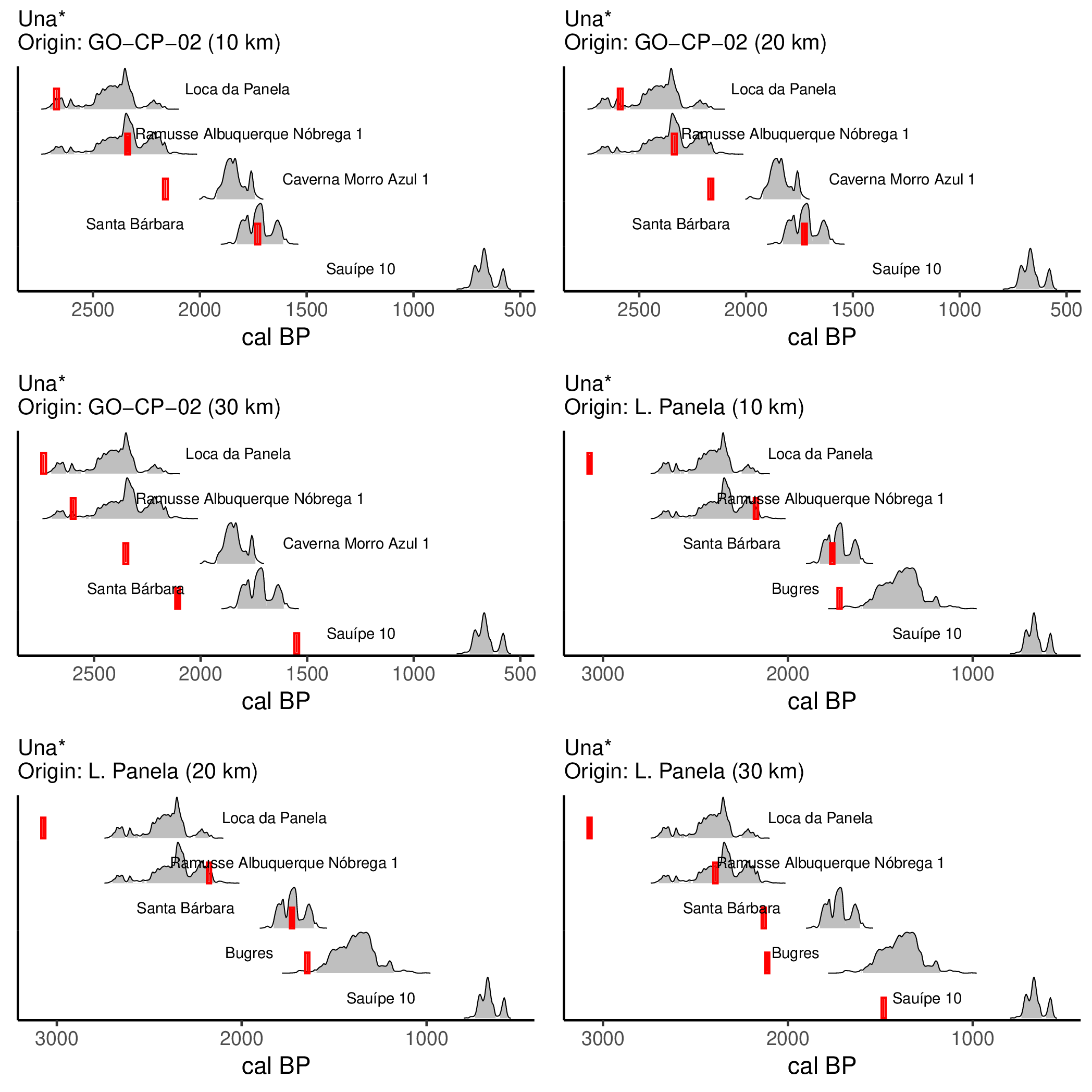

Supplement: S16 Fig — Shaded areas are the 2σ ranges of the calibrated probability densities of 14C archaeological dates, accompanied by the respective site name. Red bars represent the simulated arrival time. *Models executed without considering the earliest date for Gruta do Gentio II. (TIFF) [file pone.0232367.s019.tiff]
