# Supplementary material for: Archaeological expansions in tropical South America during the late Holocene: Assessing the role of demic diffusion
Source: PLoS One. 2020 Apr 27;15(4):e0232367. doi: 10.1371/journal.pone.0232367 (PMC7185720; doi:10.1371/journal.pone.0232367)
Supplement: S1 Appendix — (PDF) [file pone.0232367.s020.pdf]

```python
import csv
import math
import numpy as np
import os
import pyproj
import time


# Base layers are in Albers Equal Area Conical for South America.
albers = pyproj.Proj("+proj=aea +lat_1=-5 +lat_2=-42 +lat_0=-32 +lon_0=-60 \
                     +x_0=0 +y_0=0 +ellps=aust_SA +towgs84=-57,1,-41,0,0,0,0 \
                     +units=m +no_defs")


def get_dates(name):
    dates = {}
    for filename in os.listdir('./dates/{}'.format(name)):
        global x, y
        new_dates = {}
        with open('./dates/{}/{}'.format(name, filename)) as csv_file:
            csv_reader = csv.reader(csv_file, delimiter=',')
            line_count = 0
            for row in csv_reader:
                if line_count == 1:
                    x, y = to_canvas(float(row[1]), float(row[2]))
                    new_dates[(x, y)] = {}
                elif line_count > 1:
                    new_dates[(x, y)][int(row[1])] = float(row[2])
                line_count += 1
            dates.update(new_dates)
    return dates


def to_lonlat(coords):
    x, y = coords
    lon, lat = albers(x, y, inverse=True)
    return lon, lat


def transform_coords(coords):
    x, y = coords
    x_m = -2985163.8955 + (x * 10000)
    y_m = 5227968.786 - (y * 10000)
    return x_m, y_m


def to_canvas(x, y):
    x_canvas = int((albers(x, y)[0] + 2985163.8955) / 10000)
    y_canvas = int((5227968.786 - albers(x, y)[1]) / 10000)
    return x_canvas, y_canvas


class Village:
    """
    Class used to represent a village in the model.
    """

    def __init__(self, _id, model, coords, breed, start_date, k,
                 fission_threshold, catchment, leap_distance, permanence,
                 tolerance):

        self._id = _id
        self.active = True

        # Basic settings
        self.model = model
        self.coords = coords
        self.breed = breed
        self.start_date = start_date

        # Demographic parameters
        self.r = 0.025
        self.k = k
        self.total_k = 0
```

```python

        # Initialize village at saturation point
        self.population = fission_threshold

        # Territory and movement parameters
        self.catchment = catchment
        self.fission_threshold = fission_threshold
        self.leap_distance = leap_distance

        # Start with no land
        self.land = []

        # Keep track of permanence
        self.permanence = permanence
        self.time_here = 0

        self.tolerance = tolerance

    def get_neighborhood(self, radius):
        """
        Returns all the cells within a given radius from the
        village.
        """
        neighborhood = {(x, y): self.model.grid[(x, y)]
                        for x in range(self.coords[0] - radius,
                                       self.coords[0] + radius + 1)
                        for y in range(self.coords[1] - radius,
                                       self.coords[1] + radius + 1)
                        if (self.get_distance((x, y)) <= radius and
                            x >= 0 and y >= 0)}
        return neighborhood

    def get_neighbors(self, radius):
        """
        Returns the ids of all other villages within a given
        radius of the village.
        """
        neighbors = []
        neighborhood = self.get_neighborhood(radius)
        for cell in neighborhood:
            if (neighborhood[cell]['agent'] and
                    neighborhood[cell]['agent'] != self._id):
                neighbors.append(neighborhood[cell]['agent'])
        return neighbors

    def get_destinations(self, distance):
        """
        Returns all the cells that are at a given distance from the
        village.
        """
        destinations = {(x, y): self.model.grid[(x, y)]
                        for x in range(self.coords[0] - distance,
                                       self.coords[0] + distance + 1)
                        for y in range(self.coords[1] - distance,
                                       self.coords[1] + distance + 1)
                        if (self.get_distance((x, y)) == distance and
                            x >= 0 and y >= 0)}
        return destinations

    def get_empty_destinations(self, distance, pioneer=False):
        """
        Returns all cells at a given distance that are not owned.
        If in pioneer mode, restricts the search to cells that have
        never been claimed.
        """
        destinations = self.get_destinations(distance)
        if pioneer:
            available_destinations = {cell: destinations[cell][self.breed]
                                      for cell in destinations
                                      if not destinations[cell]['owner']
                                      and (destinations[cell][self.breed] >=
                                           self.tolerance)
                                      and not destinations[cell]['arrival_time']}
        else:
```

```
            available_destinations = {cell: destinations[cell][self.breed]
                                      for cell in destinations
                                      if not destinations[cell]['owner']
                                      and (destinations[cell][self.breed] >=
                                           self.tolerance)}
        return available_destinations

    def get_distance(self, next_coords):
        """
        Returns the distance (in cells) from the village to a pair of
        coordinates.
        """
        x, y = self.coords
        next_x, next_y = next_coords
        return round(math.hypot((next_x - x), (next_y - y)))

    def grow(self):
        """
        Population grows exponentially. Update land is called to add
        new cells in case population is above K.
        """
        self.population += round(self.r * self.population)
        self.update_land()

    def update_land(self):
        """
        Calculates total K from all cells owned by the village. In case
        population exceeds total K, tries to add new cells. If
        population is still beyond K after adding all available cells,
        population is reduced back to total K and the village becomes
        inactive.
        """
        while self.population > self.total_k:
            # Cells within catchment that are not owned.
            territory = self.get_neighborhood(self.catchment)
            free_land = {cell: territory[cell][self.breed]
                         for cell in territory if not territory[cell]['owner']
                         and territory[cell][self.breed] >= self.tolerance}

            if free_land:
                # Choose cell with highest suitability.
                new_land = max(free_land, key=free_land.get)
                self.claim_land(new_land)

            else:
                self.population = self.total_k
                self.active = False

    def claim_land(self, coords):
        """
        Claims a cell for the village, updates total carrying capacity
        and records the simulated date.
        """

        self.model.grid[coords]['owner'] = self._id
        self.land.append(coords)

        self.total_k = self.k * len(self.land)

    def record_date(self):
        neighborhood = self.get_neighborhood(self.catchment)
        for cell in neighborhood:
            if not self.model.grid[cell]['arrival_time']:
                self.model.grid[cell]['arrival_time'] = self.model.bp

    def check_fission(self):
        """
        If population is above fission threshold and there are
        available cells outside its catchment, the village fissions and
        the daughter village moves away. If there are no empty cells
        but leapfrogging is allowed, another search is performed for
        leap distance.
        """
        if self.population >= self.fission_threshold:
```

```python
            empty_land = self.get_empty_destinations(self.catchment * 2)
            neighbors = self.get_neighbors(self.catchment * 2)
            if empty_land and len(neighbors) < 6:
                new_village = self.fission()
                self.model.agents[new_village._id] = new_village
                new_village.move(empty_land)

            elif self.leap_distance:
                distant_land = self.get_empty_destinations(self.leap_distance,
                                                           pioneer=True)

                # Only perform leapfrogging if attractiveness of the
                # destination is higher than current cell.
                if (distant_land and max(distant_land.values()) >
                        self.model.grid[self.coords][self.breed]):
                    new_village = self.fission()
                    self.model.agents[new_village._id] = new_village
                    new_village.move(distant_land)

    def fission(self):
        """
        A new village is created with the same attributes as the parent
        village and half its population.
        """
        new_village = Village(self.model.next_id(), self.model, self.coords,
                              self.breed, self.start_date, self.k,
                              self.fission_threshold, self.catchment,
                              self.leap_distance, self.permanence,
                              self.tolerance)
        self.population //= 2
        new_village.population = self.population
        return new_village

    def move(self, neighborhood):
        """
        Moves the village to the cell with highest suitability in a
        given neighborhood. After moving, the village claims cells
        according to the population size.
        """
        new_home = max(neighborhood, key=neighborhood.get)
        if self.model.grid[self.coords]['agent'] == self._id:
            self.model.grid[self.coords]['agent'] = 0
        self.coords = new_home
        self.model.grid[new_home]['agent'] = self._id
        self.record_date()
        self.claim_land(new_home)
        self.update_land()

    def abandon_land(self):
        """
        Release ownership of cells.
        """
        for cell in self.land:
            self.model.grid[cell]['owner'] = 0
        self.land = []

    def check_move(self):
        """
        If settled beyond maximum permanence time in a given location,
        the village searches for available cells beyond its catchment
        to move. If no cells are available but leapfrogging is allowed,
        another search is performed for leap distance.
        """
        if self.time_here >= self.permanence:
            empty_land = self.get_empty_destinations(self.catchment * 2)

            if empty_land:
                self.abandon_land()
                self.move(empty_land)
                self.time_here = 0

            else:
                self.time_here += 1
```

```
        else:
            self.time_here += 1

    def step(self):
        if self.active:
            self.grow()
            self.check_fission()
            self.check_move()


class Model:
    def __init__(self, params):

        self.width = 638
        self.height = 825

        self.current_id = 0

        self.agents = {}
        self.grid = {}

        # Model parameters
        self.params = params

        # Start at earliest date in the model
        self.start_date = self.params[2]
        self.bp = self.start_date

        # Layers to keep track of agents, land ownership and dates
        # of arrival for each culture.
        for y in range(self.height):
            for x in range(self.width):
                self.grid[(x, y)] = {'agent': 0,
                                     'owner': 0,
                                     'arrival_time': 0}

        # Add layers with ecological niche of each culture.
        breed_name = self.params[1]
        layer = np.loadtxt('./layers/{}.asc'.format(breed_name), skiprows=6)
        for y in range(self.height):
            for x in range(self.width):
                self.grid[(x, y)][breed_name] = layer[y, x]
                # Prevent water cells from being settled.
                if layer[y, x] == -9999:
                    self.grid[(x, y)]['owner'] = -1

        self.setup_agents()

    def next_id(self):
        """
        Generates continuous unique id values for agents.
        Start at 1.
        """
        self.current_id += 1
        return self.current_id

    def setup_agents(self):
        """
        Create a village for each culture, add land to their territory
        and record their start dates (not current year).
        """
        village = Village(self.next_id(), self, *self.params)
        self.agents[village._id] = village
        self.grid[village.coords]['agent'] = village._id
        village.claim_land(village.coords)
        village.record_date()

    def eval(self):
        """
        Returns a score from 0 to 1 of model fitness based on match
        with archaeological dates.
        """
        total_score = 0

```

```
        breed_name = self.params[1]
        dates = get_dates(breed_name)

        for coords in dates:
            score = 0
            sim_date = self.grid[coords]['arrival_time']
            if sim_date and sim_date in dates[coords]:
                # Normalize probability distribution
                score += (dates[coords][sim_date] /
                          max(dates[coords].values()))

            total_score += score

        return total_score / len(dates)

    def step(self):
        agent_list = list(self.agents.keys())
        for _id in agent_list:
            self.agents[_id].step()
        self.bp -= 1
```
