# Supplementary material for: Archaeological expansions in tropical South America during the late Holocene: Assessing the role of demic diffusion
Source: PLoS One. 2020 Apr 27;15(4):e0232367. doi: 10.1371/journal.pone.0232367 (PMC7185720; doi:10.1371/journal.pone.0232367)
Supplement: S2 Appendix — (PDF) [file pone.0232367.s021.pdf]

```python
import copy as cp
import multiprocessing as mp
import numpy as np
import pickle
from time import time

from model import Model


def run_model(params):
    init_time = time()

    params.insert(0, to_canvas(-50.5414, -5.8059))                              # coordinates of origin
    params.insert(1, 'tupi')                                                    # name
    params.insert(2, 2411)                                                      # start date bp
    params.append(0.442)                                                        # maxent threshold

    new_model = Model(params=params)

    for i in range(new_model.start_date - 500):
        new_model.step()
        # Abort if execution time is too long and assign
        # worst possible score to avoid passing to next
        # generation.
        if time() - init_time >= 600:
            return 0

    result = new_model.eval()
    return result


# carrying capacity
class Gen_01:
    def initialize(self):
        self.value = int(np.random.uniform(20, 100, 1)[0])
    def mutation(self):
        if 20 < self.value < 100:
            aux = self.value + (int(np.random.uniform(-40, 40, 1)[0]) or
                                np.random.choice((-1, 1)))
        # If value is already minimum, can only increase;
        # if maximum, can only decrease.
        elif self.value == 20:
            aux = self.value + int(np.random.uniform(1, 40, 1)[0])
        elif self.value == 100:
            aux = self.value - int(np.random.uniform(1, 40, 1)[0])

        if aux > 100:
            self.value = 100
        elif aux < 20:
            self.value = 20
        else:
            self.value = aux


# fission
class Gen_02:
    def initialize(self):
        self.value = int(np.random.uniform(50, 300, 1)[0])
    def mutation(self):
        if 50 < self.value < 300:
            aux = self.value + (int(np.random.uniform(-100, 100, 1)[0]) or
                                np.random.choice((-1, 1)))
        # If value is already minimum, can only increase;
        # if maximum, can only decrease.
        elif self.value == 50:
            aux = self.value + int(np.random.uniform(1, 100, 1)[0])
        elif self.value == 300:
            aux = self.value - int(np.random.uniform(1, 100, 1)[0])

        if aux > 300:
            self.value = 300
        elif aux < 50:
            self.value = 50
        else:
```

```python
            self.value = aux


# catchment
class Gen_03:
    def initialize(self):
        self.value = np.random.choice((1, 2, 3), 1)[0]
    def mutation(self):
        if self.value == 2:
            self.value += np.random.choice((-1, 1), 1)[0]
        else:
            self.value = 2


# leapfrogging
class Gen_04:
    def initialize(self):
        if 0.5 > np.random.uniform(0, 1, 1)[0]: # 50% prob to set 0
            self.value = 0
        else:
            self.value = int(np.random.uniform(15, 25, 1)[0])
    def mutation(self):
        if 0.25 > np.random.uniform(0, 1, 1)[0]: # 25% prob to set 0
            self.value = 0
        else:
            if self.value == 0:
                self.value = 15
            else:
                if 15 < self.value < 25:
                    aux = self.value + (int(np.random.uniform(-10, 10, 1)[0]) or
                                        np.random.choice((-1, 1)))
                # If value is already minimum, can only increase;
                # if maximum, can only decrease.
                elif self.value == 15:
                    aux = self.value + int(np.random.uniform(1, 10, 1)[0])
                elif self.value == 25:
                    aux = self.value - int(np.random.uniform(1, 10, 1)[0])

                if aux > 25:
                    self.value = 25
                elif aux < 15:
                    self.value = 15
                else:
                    self.value = aux


# permanence
class Gen_05:
    def initialize(self):
        self.value = int(np.random.uniform(10, 30, 1)[0])
    def mutation(self):
        if 10 < self.value < 30:
            aux = self.value + (int(np.random.uniform(-10, 10, 1)[0]) or
                                np.random.choice((-1, 1)))
        # If value is already minimum, can only increase;
        # if maximum, can only decrease.
        elif self.value == 10:
            aux = self.value + int(np.random.uniform(1, 10, 1)[0])
        elif self.value == 30:
            aux = self.value - int(np.random.uniform(1, 10, 1)[0])

        if aux > 30:
            self.value = 30
        elif aux < 10:
            self.value = 10
        else:
            self.value = aux


# genome object
class Genome:
    def __init__(self, genes):
        self.genes = genes
        self.fitness = 'OFF'
```

```python
    def initialize(self):
        self.genes = [Gen_01(), Gen_02(), Gen_03(), Gen_04(), Gen_05()]
        for i in self.genes:
            i.initialize()
    def mutation(self, prob):
        if prob > np.random.uniform(0, 1, 1)[0]:
            mpoint = np.random.choice(range(0, len(self.genes)), 1)[0]
            self.genes[mpoint].mutation()
            self.fitness = 'OFF'
    def genes_values(self):
        return [i.value for i in self.genes]


# algorithm object
class GA:
    def __init__(self, n_pop, n_select, n_elit, prob_cross, prob_mut, max_it):
        # algorithm parameters
        self.n_pop = n_pop
        self.n_select = n_select
        self.n_elit = n_elit
        self.prob_cross = prob_cross
        self.prob_mut = prob_mut
        self.max_it = max_it

    # initialize
    def initialize(self):
        self.population = [Genome(0) for i in range(self.n_pop)]
        for i in self.population:
            i.initialize()

    # compute fitness
    def get_fitness(self):
        pop_subset = [p for p in self.population if isinstance(p.fitness, str)]
        pop_genes = [i.genes_values() for i in pop_subset]
        pool = mp.Pool(10)                                                                    # n cores
        fitness = np.array(pool.map(run_model, pop_genes))
        pool.close()
        for i in range(len(pop_subset)):
            pop_subset[i].fitness = fitness[i]
        return np.array([p.fitness for p in self.population])

    # best individuals
    def get_parents(self, fitness):
        parents = [self.population[i] for i in np.argsort(-fitness)[range(self.n_select)]]
        return parents

    # crossover
    def do_crossover(self, parents):
        crossovers = []
        ind1 = 0
        ind2 = 1
        while len(crossovers) < self.n_pop:
            parent1 = parents[ind1]
            parent2 = parents[ind2]
            if self.prob_cross > np.random.uniform(0, 1, 1)[0]:
                pt = np.random.choice(range(1, len(parent1.genes)), 1)[0]
                child1 = Genome(parent1.genes[0:pt] + parent2.genes[pt:len(parent2.genes)])
                child2 = Genome(parent2.genes[0:pt] + parent1.genes[pt:len(parent1.genes)])
                crossovers.append(cp.deepcopy(child1))  # deepcopy
                crossovers.append(cp.deepcopy(child2))  # deepcopy
            else:
                crossovers.append(cp.deepcopy(parent1)) # deepcopy
                crossovers.append(cp.deepcopy(parent2)) # deepcopy
            ind1 = int(np.where(ind1 != len(parents)-1, ind1 + 1, 0))
            ind2 = int(np.where(ind2 != len(parents)-1, ind2 + 1, 0))
        return crossovers

    # mutation and set new population
    def do_mutation(self, crossovers):
        for i in crossovers:
            i.mutation(self.prob_mut)
        return crossovers

    # evolve loop
```

```python
    def evolve(self):
        FILE = open('GA.pyData', 'wb')
        for it in range(self.max_it):
            fitness = self.get_fitness()
            parents = self.get_parents(fitness)
            crossovers = self.do_crossover(parents)
            mutated = self.do_mutation(crossovers)
            self.population = mutated + cp.deepcopy([parents[i] for i in
                                                    range(self.n_elit)])
            if it % 10 == 0:
                print('\n it: ' + str(it + 1) + ' writing ...\n')
                pickle.dump(self, FILE)
                FILE.close()
            print('\n========== Iteration: ' + str(it + 1) + ' ===========\n')
            for i in parents[0:5]:
                i.Print()

def main():
    my_ga = GA(100, 40, 5, 0.8, 0.2, 21)
    my_ga.initialize()
    my_ga.evolve()


if __name__ == "__main__":
    main()
```
