# Supplementary material for: Archaeological expansions in tropical South America during the late Holocene: Assessing the role of demic diffusion
Source: PLoS One. 2020 Apr 27;15(4):e0232367. doi: 10.1371/journal.pone.0232367 (PMC7185720; doi:10.1371/journal.pone.0232367)
Supplement: Attachment — Submitted filename: response.docx [file pone.0232367.s022.docx]

**Response to Reviewer #2**

In what follows, the reviewer’s comments are given in black, and our responses appear in blue.

---

Reviewer #2: This is a comprehensive review of data dispersed in different sources, some of them unpublished reports or local publications in Portuguese and Spanish. I commend the authors for their effort in bringing all this information together. It is also a manuscript that addresses the old debate of correlating language and archaeological patterns in lowland South America. The major difference here is that instead of focusing in single language families, the authors employ a continental-scale approach with different language families and archaeological units (phases, traditions etc.). They do so by building simulations from the compiled data set.

I personally thing this is an interesting approach, although I confess I have not much to say about the logic and the procedures that inform the simulation. I also feel that, as much as I am in sympathy with the authors and their effort here, the paper has some problems that need to be dealt with if gets published. Some of these problems are interpretative while others have to do with the data. While the former relate to personal perspectives to approach the data, and are hence minor, the latter need to be addressed or better qualified by the authors.

The authors perspective is that “the vast territories occupied by the largest language families of South America … suggest(…) that sizable demographic expansions may have taken place in pre-Columbian times. Further support is offered by the distribution of 52 archaeological cultures that emerged and expanded over the last 5000 years”. The authors propose that some of theses expansions may result from demographic growth following the adoption of agriculture. To do so they examined four of such language dispersions and correlate them to archaeological patterns to run their simulations and test such assumption.

The four language families addressed in the manuscript – Arawak, Tupi-Guarani, Carib and Ge – have indeed continental scale distributions, mostly the former two. However, such distribution does not need to be explained from a demic diffusion perspective. The idea of correlating demographic expansions with the adoption of agriculture in lowland South America is even older than the concept of demic diffusion and can be credited to Donald Lathrap as the authors rightly acknowledge. That said, the authors themselves concede that the reasoning that provided the backing for such hypotheses – the premise that slash-and-burn itinerant manioc cultivation was a prevailing pattern in the past – has been falsified by recent research. I am in agreement with them when they propose the concept of policulture agroforestry as better way to explain such ancient productive systems, which would have been characterized by the cultivation of short and long-cycle crops, including trees. Davis Harris (2003: 31-32) proposed that “the nutritional potential and expansion capacity of EASs (early agricultural systems) were strongly influenced by the presence or absence of domestic herd animals, cereals, pulses (herbaceous legumes), tree and root crops… Tree crops are nutritionally valuable, especially as a source of vegetable oils, but because they are long-lived perennials their cultivation has been inimical to agricultural expansion. So too has been the cultivation of carbohydrate-yielding root crops, which is commonly complemented with protein obtained by fishing and hunting”. I feel this short description fits really well the case of language dispersals in tropical lowland South America and explains it better than a demic diffusion model. It would be interesting to see how such premises would build into a simulation model.

We agree with the observation that polyculture agroforestry, as practised in lowland South America, could have resulted in different expansion capacity of early agricultural systems. Citing Harris, as suggested by the reviewer, we now state that:

In fact, it has been suggested that the expansion capacity of early agricultural systems was greatly determined by the type of economic “package” - whether it included tree and root crops (as in lowland South America) or cereals and herd animals [34] (p. 4)

In addition, we specify that, although the details of the agricultural systems in question are not modelled, they are implicit:

Although the detailed dynamics of polyculture agroforestry are not explicitly modeled (which would be impractical at the spatial and temporal scales of the simulations), they are implicit in the dynamics of growth, fission and movement of the villages, as inferred from the ethnographic literature (p. 18).

In the following lines I offer specific comments:

103-104 - “The spread of Saladoid-Barrancoid material culture can be traced to the Orinoco ~4600 cal BP, expanding over most of the Amazon by ~3400-2900 cal BP, except for the southern limits of the basin, which were settled ~1000 cal BP [38,42]”.

223 - The former proposal places the origins of Saladoid ceramics ~4500 cal BP, whereas the latter delays its origins until ~2500 cal BP. The Barrancoid style, on the other hand, is commonly accepted to have begun ~3000 cal BP. Given the lack of consensus, here we maintain the long chronology following recently obtained dates for the Saladero phase, which show that the development of that ceramic style fits poorly in the short chronology [45]. Furthermore, it is easier to conciliate the long chronology with the early dates for Pocó-Açutuba sites in central and lower Amazon [21].

The above assumptions are based in the acceptance of the long-chronology for the Orinoco. The authors are aware of this, but this issue is far from settled. I personally think that a short chronology explains better the data and the patterns there. I may have not noticed, but it would be interesting to run the simulations with a short chronology for the Orinoco as well.

We have now run the regression analyses and simulations with the short chronology for the Orinoco (see new data in Fig 3, Table 2, S4 and S11 Figs, S3 Table).

In the results of the regression analyses, we have noticed that:

Overall, using the short chronology for Saladoid-Barrancoid resulted in poorer fits because many early sites with similar ages would be found at large distances from each other (p. 23).

(...)

This delay [i.e., in reaching the southern Amazon] is not observed using the short chronology for Saladoid-Barrancoid, where the problem seems to be the existence of many sites with similar early ages very far from each other, making it difficult to be modeled as a gradual expansion from a specific origin (Fig 3) (p. 24)

In the results of the simulations, we stated that:

Some of the models evaluated using the short chronology for the Orinoco tended to correctly predict arrival times in the northernmost and southernmost extremes of the Saladoid-Barrancoid expansion (S4 and S11 Figs). They failed, however, to capture the timing of the settlement in western, central and eastern Amazon, where arrival times are too late. As noticed in the results of the regression analyses, this is due to the existence of many early dates in those areas, suggesting a nearly simultaneous appearance of the Saladoid-Barrancoid phenomenon if the short chronology is to be accepted (p. 27-28)

124 – “Incised-Punctate settlement patterns and site architecture are diverse. In the Guianas coast, settlements included habitation mounds surrounded by complexes of agricultural raised fields 125 [50,51].” Here they follow the published literature, but I believe that the presence of Arauquinoid and I-P in the Guiana coast will be reviewed in the light of future research with Koriabo ceramics.

Indeed, we agree that fture revisions of the Incised-Punctate tradition may affect the plausibility of the models. Specifically for the Koriabo case, we call attention to our observation that, since the earliest dates in every region are the most important for evaluating the models, “the Koriabo culture is recent, and excluding its dates from the analysis would not substantially affect the results” (p. 11)

171 – “In spite of the distinctiveness in its settlement patterns, the Una expansion has also been sustained by an agricultural base, as evidenced by the presence of maize in storage facilities since the earliest occupations in rock shelters, together with other domesticated plant remains like manioc and beans in later periods”. I feel that the mere presence of maize in the record does not warrant associating Una expansion with farming. As the authors concede, (661) “as in the Tupiguarani case, the Una expansion is notably difficult to reproduce. Population densities were the highest of all the best models (0.77 individuals km-2 660 ) and the fission threshold (142 persons) was higher than for Saladoid-Barrancoid and Incised-Punctate (Table 4). Those parameters are in blatant disagreement with the archaeological settlement data: the earliest Una sites are occupations in rock shelters with ephemeral duration and low density of material culture [24,69,154]”. I agree with them and believe that the Una case should be removed from a demic diffusion approach.

We agree with those comments, and the revised manuscript now states clearly that:

Thus, the presence of maize and other cultivated plants in the archaeological record of the Una tradition [70-71] may not necessarily imply that agricultural products were the basis of the people’s diet and/or the drivers of its expansion, perhaps excluding this archaeological case from the expectations of the FLDH (p. 31)

The final statement reads as: (680) – “In this paper, we reevaluated late Holocene archaeological expansions in tropical South America, comparing them to cases of demic diffusion elsewhere through the analysis of spatial gradients in radiocarbon dates and by means of computer simulations. We found that some archaeological phenomena could be successfully modeled as demic waves of advance, as is the case of the Saladoid-Barrancoid and Incised-Punctate expansions, which gives further credence to their association with the spread of language families, respectively (branches of) Arawak and Carib, an association which has long been hypothesized [12,32,76]. Other dispersals, however, were more difficult to simulate as purely demographic processes. In the Tupiguarani case, the source of disagreement appears to be the lack of an identifiable spatial pattern in the distribution of 14C dates, calling for more research as well as a reappraisal of dates commonly discarded. As for the Una expansion, a strong case can be made for the role of cultural diffusion [24,54], but the reason why it has proceeded much slower than expected remains to be solved in light of recent models that suggest cultural diffusion should proceed faster than demic advance [99]. In any case, a relationship between the Una tradition and the (Macro-)Jê languages should not be discarded, since cultural diffusion could have taken place among pre-agricultural speakers of that language family – as it has, indeed, been suggested in the past [54]. Importantly, the combination of demographic processes for some cultures (Saladoid-Barrancoid, Incised-Punctate) and potentially cultural processes in others (Una) that we see in tropical South America are comparable to the Neolithic expansion in other parts of the globe [99,127] where both situations have been observed.”

Incise-Punctate is a category that probably will be abandoned in the near future as other complexes also potentially related to Carib languages, such as Koriabo, become better known. Hence, maybe the only remaining case for demic diffusion in tropical lowland South America would be Saladoid/Barrancoid/Incised Rim and Arawak. In the light of this, I suggest the authors to rephrase their main argument and structure the ms. as evidence of other processes leading to language dispersals in this part of the world.

As for the Koriabo tradition, see our response to p. 124 above. In addition, we have added the following observations to the conclusions of the paper:

Two cautionary notes, however, must be made. First, simulations using the short chronology for the Orinoco failed to model the Saladoid-Barrancoid expansion satisfactorily, mainly due to the existence of several early dates across the Amazon. If correct, these data would imply that processes other than demic diffusion were behind the dispersal of Saladoid-Barrancoid material culture – as suggested, for example, by Hornborg’s trade model [31]. Second, the Incised-Punctate models depend on the correct identification of the ceramic complexes that should be included in this broadly-defined cultural unit – a definition that may change, for example, if the Koriabo tradition is indeed shown to be an unrelated phenomenon [88]. In this work, we have followed the published literature while recognizing that future data may cast doubt on the expansion of the Incised-Punctate culture and of the Carib languages as a purely demic process (p. 31)

I believe that everyone working with this subject may agree that some form of intensification of plant cultivation or agriculture may lay behind these demographic expansions. However, the remarkable fact about tropical lowland South America is the large amount of language families/genealogical units and isolated languages found there: more than 50. Most of those families have a local geographic expression, while some of them may have a wider distribution at a sub-continental scale, such as Pano. The four families addressed in the paper are the ones with largest distribution, but instead of trying to approach those I feel that a major conceptual contribution to archaeology will emerge when one investigates the deep history of the other dozens of families with localized distributions. In other words, the major anthropological question lies in understanding why so much language diversity emerged in the lack of major geographical barriers. This is beyond the scope of this manuscript, of course, but looking at the potential role of demic diffusion tells just one side of the story and does not offer a novel theoretical or factual contribution, as the authors themselves would agree.

We agree that the level of linguistic diversity in lowland South America is remarkable. We stress that our intention with this paper is not to propose demic diffusion as the only mechanism behind the archaeological expansions in question, as we believe is clear from the results and discussion of the simulations, but that it may have played a role in some of them. We have now made a more nuanced discussion by including the following paragraphs:

At the same time, we recognize the possibility that to fully understand the majority of archaeological expansions in lowland South America it might require explanations that go beyond purely demic diffusion, especially considering the difficulties in modeling the Tupiguarani expansion or even the Saladoid-Barrrancoid expansion if the short chronology is to be accepted (p. 32)

(...)

As it has been highlighted before, lowland South America is home to considerable ethnolinguistic diversity [16], and a crucial task for future models will be to assess the role of demic and cultural processes in the emergence of such diversity, beyond the four expansions considered in this work (p. 32)
